# Mid-Term Outcomes after Arthroscopic “Tear Completion Repair” of Partial Thickness Rotator Cuff Tears

**DOI:** 10.3390/medicina57010074

**Published:** 2021-01-17

**Authors:** Giuseppe Fama, Jacopo Tagliapietra, Elisa Belluzzi, Assunta Pozzuoli, Carlo Biz, Pietro Ruggieri

**Affiliations:** 1 Orthopaedic Clinic, UOC Azienda Ospedaliera of Padova, 35128 Padova, Italy; giuseppe.fama54@gmail.com (G.F.); pietro.ruggieri@unipd.it (P.R.); 2 Orthopaedic and Traumatologic Clinic, Department of Surgery, Oncology and Gastroenterology, University of Padova, 35128 Padova, Italy; tagliapietra.j@gmail.com (J.T.); assunta.pozzuoli@unipd.it (A.P.); 3 Musculoskeletal Pathology and Oncology Laboratory, Orthopaedic and Traumatologic Clinic, Department of Surgery, Oncology and Gastroenterology, University of Padova, 35128 Padova, Italy

**Keywords:** partial-thickness rotator cuff tears, critical zone, arthroscopic tear completion repair, microfractures

## Abstract

*Background and Objectives*: Different arthroscopic procedures are used for partial-thickness rotator cuff tears (PT-RCTs), but there is still no evidence on the superiority of one procedure over the other. The aim of this study was to evaluate the clinical outcomes and the rate of complications of a tear completion repair (TCR) technique. *Materials and Methods:* Patients who had undergone arthroscopic TCR technique for PT-RCTs with a follow-up of at least 2-years after surgery were included. The TCR technique involved the removal of the “critical zone” and creating microfractures to biologically support tendon healing. Functional outcomes were assessed prospectively by the Constant score (CS) and active and passive range of movement (ROM). Pain and patient satisfaction were measured using a visual analog scale (VAS). Complication rates were recorded, and tendon integrity was assessed with magnetic resonance imaging (MRI) or ultrasound performed at least 2-years after surgery. *Results:* Eighty-seven patients with a median age of 57 years were followed-up for a median of 5 years. The CS score improved from 53.5 preoperatively to 94.0 postoperatively (*p* < 0.001). Median VAS score decreased from 8.6 to 1.0 (*p* < 0.0001). Median patient satisfaction was 9.3. The overall complication rate was 14.9%. *Conclusions:* Patients with PT-RCTs of the supraspinatus tendon treated by the TCR technique with “critical zone” removal and biological stimulation by microfractures showed good functional results with excellent strength recovery, a high degree of patient satisfaction, and resolution of painful symptoms at mid-term follow-up.

## 1. Introduction

Partial-thickness rotator cuff tears (PT-RCTs) represent a common cause of shoulder pain and disability in the adult population [1,2]. PT-RCTs are more common than full-thickness tears (FT-RCTs) [3], and the supraspinatus tendon is most usually involved [4]. PT-RCTs can involve also to the subscapularis tendon [5]. The prevalence of PT-RCTs ranges from 13% to 32%, and it is strongly related to patient age [6,7,8] with a linear increase after the fifth decade of life [9].

Pathogenesis of PT-RCTs is related to intrinsic and/or extrinsic factors, the former being most common. Intrinsic factors include tendon changes due to traumatic or, most often, degenerative causes. Extrinsic factors originate from the tissues surrounding the cuff and include anatomic variants and, above all, subacromial impingement (mainly bursal side lesion) [10,11] and posterosuperior glenoid impingement [12]. Certainly, the instability of the long head of the biceps tendon (LHBT), which passes underneath the rotator cuff (RC) to its anchor on the superior labrum and glenoid, is one important trigger factor in gradual RC pathology [13,14]. Pathogenesis differs according to age: postero-superior glenoid impingement represents the main cause of PT-RCT in young athletes (under 30 years of age) [12], post-traumatic tears are more frequent in patients around 40–50 years and degenerative ones in patients over 60 [15,16].

**Figure 1:**
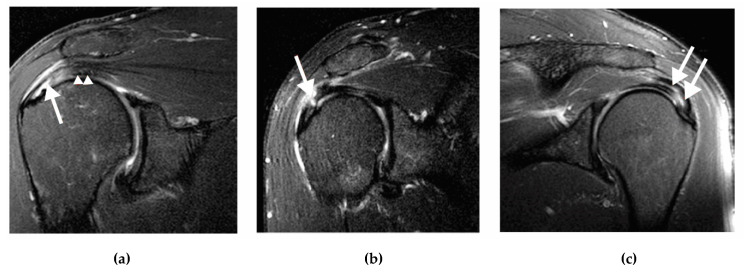
Coronal magnetic resonance imaging (MRIs) showing the three types of partial thickness of ST tears. (**a**) articular insertional tear (arrow), ST (arrow head); (**b**) bursal tear (arrow); (**c**) intratendinous tear (arrows). MRI, magnetic resonance imaging; ST, supraspinatus tendon.

PT-RCTs can be classified on the basis of their anatomical location (articular, bursal, or intratendinous) and size (i.e., grade 1, <3 mm; grade 2, 3–6 mm; grade 3, >6 mm) according to Ellman (Figure 1) [1].

Articular-sided PT-RCT arises from degenerative changes associated to hypo-vascularisation and loss of elasticity of the collagen fibers of the critical zone of the rotator cuff (RC) tendon [17]. Bursal-sided PT-RCT is associated to thickening or ossification of the attachment of the coracoacromial ligament [17,18]. Intratendinous tears seem to be mainly related to mechanical shearing stresses between the superficial and deep layers. They differ from chronic tendinopathies and from full-thickness tears of RC, but there is no consensus in the literature concerning their etiology [19,20,21].

PT-RCTs can be treated conservatively or surgically. Conservative treatment includes rest from exacerbating activities, physical therapy, non-steroidal anti-inflammatory (NSAID) medications, intra-articular, or subacromial corticosteroid injections [16,22,23].

Absolute indications for surgical treatment have not been established until now, but many authors advocate surgery for active adults with tears of more than 50% of tendon thickness, lesions that extend beyond 2 cm in diameter, and in the case of failure of conservative treatment [16,22,23,24].

Currently, the main arthroscopic procedures are rotator cuff debridement with or without acromioplasty, transtendon repair (TTR), and tear completion repair (TCR). Some authors advise debridement with or without acromioplasty in cases of a symptomatic lesion involving less than 50% of tendon thickness (Ellman grade I and II ruptures) [25,26] and not responding to conservative treatment [23,25,26]. Repair techniques (TTR and TCR) are generally preferred for larger lesions (Ellman grade III lesions) [23]. To date, there is still no evidence on the superiority of one procedure over the other [23,27,28], and reports on the outcomes of the TCR technique are lacking [29].

In this study, we report our experience with a TRC technique that involves the completion of the lesion by removing the pathological area of the tendon corresponding to the “critical zone,” a small area of diminished vascularisation about 10 mm from the proximal insertion of the supraspinatus tendon, described for the first time by Codman in 1934 [30]. Additionally, microfractures are carried out to support tendon healing with biological stimulation and stable fixation with single row repair.

The aim of this study was to evaluate the clinical outcomes of patients undergoing the TCR technique described here for a PT-RCT. The hypothesis was that the patients would have an improvement in clinical outcomes and low complication rate.

## 2. Materials and Methods

### 2.1. Study Population

Between January 2007 and December 2016, 122 consecutive patients with a diagnosis of isolated PT-RCT of supraspinatus tendon who underwent arthroscopic repair using the TCR technique performed by a single senior surgeon were retrospectively enrolled. The study was performed in accordance with the ethical standards of the 1964 Declaration of Helsinki as revised in 2000. The Local Ethical Committee approved the study (Prot. N. 0064488, 10 October 2019).

The inclusion criteria were (1) a PT-RCT of isolated supraspinatus tendon diagnosed by preoperative magnetic resonance imaging (MRI) and confirmed intra-operatively, (2) previous failed conservative treatment (physical therapy for at least 4 months, non-steroidal anti-inflammatory (NSAID) medications, intra-articular or subacromial corticosteroid injections), (3) arthroscopic TCR, (4) postoperative MRI or ultrasound to confirm the integrity of RCT repair, and (5) a follow-up at a minimum of 2 years postoperatively. The exclusion criteria were (1) previous surgery such as RC repair on the same shoulder, (2) active frozen shoulder, (3) infections, and (4) calcified tendinitis of supraspinatus tendon.

### 2.2. Surgical Technique

**Figure 2:**
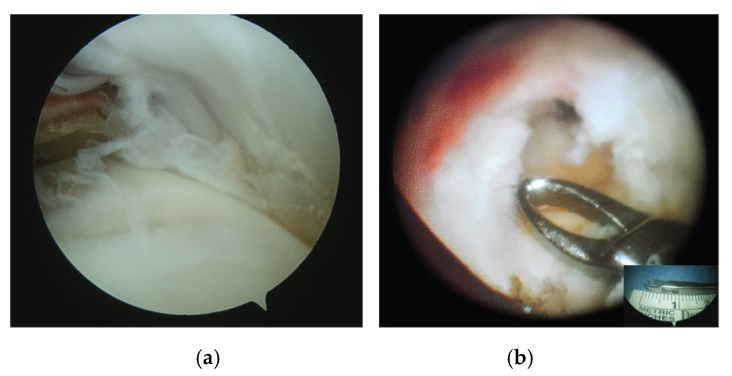
(**a**) Arthroscopic view of an endoarticular tear of the ST (posterior portal). (**b**) Arthroscopic view of the bursal surface of ST after the removal of the “critical zone,” a small area of decreased vascularity about 10 mm from the proximal insertion of the ST (lateral portal). Insert shows the size of the forceps’ edge (about 10 mm); ST, supraspinatus tendon.

The procedures were performed by a single senior surgeon with the patient in a beach-chair position under general anaesthetic and interscalene block. The visual assessment of the glenohumeral joint was done by inserting the arthroscope HD4300 (ConMed Corporation Utica, NY, USA) through the posterior portal. An anterior intra-articular portal was then performed lateral to the coracoid process with an inside-out technique. The partial tear was identified in the articular side of the RC (Figure 2a).

The lesion was then marked with a spinal needle introduced by the lateral portal passing the tendon’s tear and reaching the articular surface. In case of an intratendinous tear, using a spinal needle guide, the tendon lesion was recognised as the ‘‘weakest’’ or ‘‘more lax’’ area, while the interface between tendon and bone was normal [19]. Then, based on the needle guide, a shaver was introduced through the lateral portal to complete the lesion.

**Figure 3:**
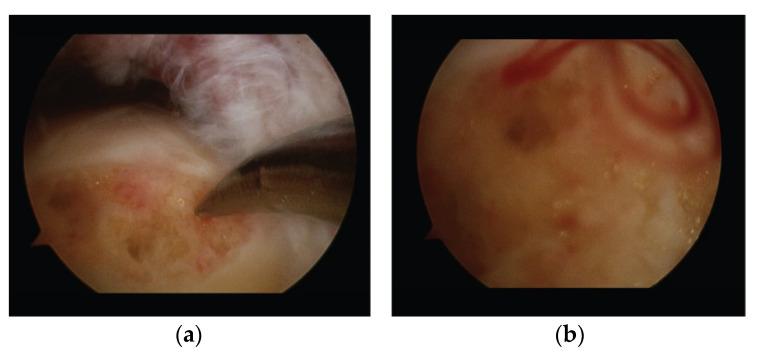
(**a**) Arthroscopic view of microfractures at the footprint (lateral portal). (**b**) When the pump is turned off, the bone marrow flows out of the microfractures to form the “Crimson duvet” containing mesenchymal stem cells, platelets, growth factors, and vascular elements that biologically support tendon healing.

Both bursal and articular tears were debrided and converted to a full-thickness tear by the removal of the pathological tendon area (on average 8–12 mm), corresponding to the “critical zone” (Figure 2b) until healthy tendon was visible. The residual fibrils of the tendon on the footprint were also debrided to expose cortical bone. A cancellous bone bed was prepared using a burr. Then, small microfractures (1.5–2 mm) of the cortical and cancellous bone of the footprint were performed in order to facilitate tendon healing (Crimson duvet) [31] (Figure 3).

A single-row tendon repair technique was then carried out using a 5.0 mm titanium suture anchor (II, Stryker European Operations B.V., Amsterdam, Netherlands) positioned on the lateral aspect of the cancellous bed through an additional transdeltoid portal performed just lateral to the acromion. Sutures were passed through the tendon in a simple vertical fashion using a birdbeak arthroscopy suture-passer device (Mitek, Johnson & Johnson, New Brunswick, NJ, USA) or a clever hook suture retriever (Mitek, Johnson & Johnson, New Brunswick, NJ, USA).

In all cases of tenosynovitis, dislocation, subluxation or LHBT tears in active young patients, a tenodesis was performed using a suture anchor (Stryker European Operations B.V., Amsterdam, Netherlands) positioned proximally to the bicipital groove. In patients from 50 to 65 years of age, a tenodesis to the coracohumeral ligament was carried out with a braided non-absorbable polyester suture (Arthrex TigerWire, Naples, FL, USA) using a percutaneous intra-articular transtendon technique (Sekiya, Baumgarten). In older patients, a tenotomy of LHBT was performed [32,33]. In all patients, a debridement of the subacromial bursa was performed, followed by an antero-inferior acromioplasty according to Neer [34].

### 2.3. Pre- and Postoperative Management

Pre- and postoperative clinical data were evaluated by the Constant Score (CS), range of motion (ROM), and pain. To evaluate ROM, active and passive forward flexion, abduction, external (ER) and internal rotation (IR) were assessed in supine position at 0° (ER 1 and IR 1) and 90° (ER 2 and IR 2) of abduction, measured using a goniometer. Pain was assessed with a visual analog scale score (VAS) (range 0–10). Clinical outcomes were assessed at least 2 years after surgery. In addition, patient satisfaction was evaluated using a VAS satisfaction scale (range 0–10).

**Figure 4:**
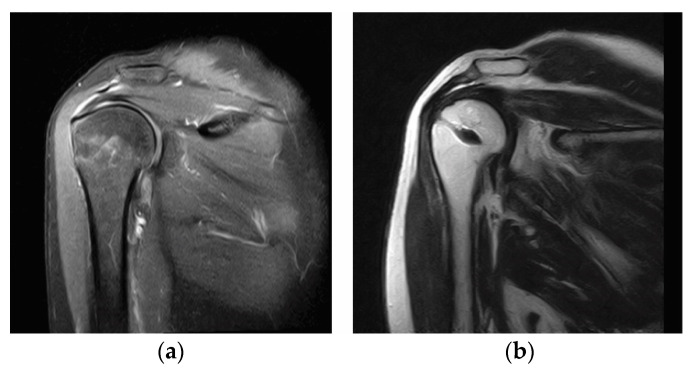
(**a**) Coronal MRI showing a partial tear involving the bursal side of the supraspinatus tendon. (**b**) Coronal MRI showing tendon healing after tear repair with the tear completion repair (TCR) technique. MRI, magnetic resonance imaging; TCR, tear completion repair.

Postoperative standard x-rays were performed to confirm the correct position of the suture anchor. Tendon integrity was assessed with MRI or ultrasound (performed at follow-up of least 2 years) (Figure 4) and classified depending on the footprint coverage: complete healing (total coverage of the footprint), complete retear (no coverage), and partial retear (partial coverage) [35,36].

After surgery, an abduction sling was applied for 5 weeks. During this period, patients were allowed to perform gentle passive movements on the scapular plane and active movements of elbow and wrist. After 5 weeks, the sling was removed in order to gradually increase passive movement as tolerated and avoiding external and internal rotations, and to start gentle active movements. After 16–18 weeks, patients were allowed to perform rotational strengthening exercises. During the final phase (about 20 weeks), patients progressed to advanced exercises and patient-specific activities aimed at increasing motion velocity. Sports and high-demand activities were not allowed until 6–8 months post-surgery.

### 2.4. Statistical Analysis

The Shapiro-Wilk test was used to determine whether quantitative data were distributed normally. The Wilcoxon rank-sum test was used to compare preoperative with postoperative VAS, Constant Score and ROM of the overall cohort. The Kruskal-Wallis test was applied to compare quantitative variables between the patients divided into three groups according to the Ellman classification. A chi-square (χ^2^) test was performed to compare categorical variables. BMI between the three groups was analysed by ANOVA test because the data were normally distributed.

The mean ± standard deviation (SD) and median with range were provided for continuous variables, while frequency and percentage distribution were reported for categorical variables. A *p*-value less than 0.05 was considered statistically significant.

All statistical analyses were carried out with the commercial software “Statistical Package for the Social Sciences” (SPSS version 25.0 for Windows, IBM, Armonk, NY, USA).

## 3. Results

Between January 2007 and December 2016, 122 patients underwent arthroscopic repair for an isolated PT-RCT of the supraspinatus tendon.

**Figure 5:**
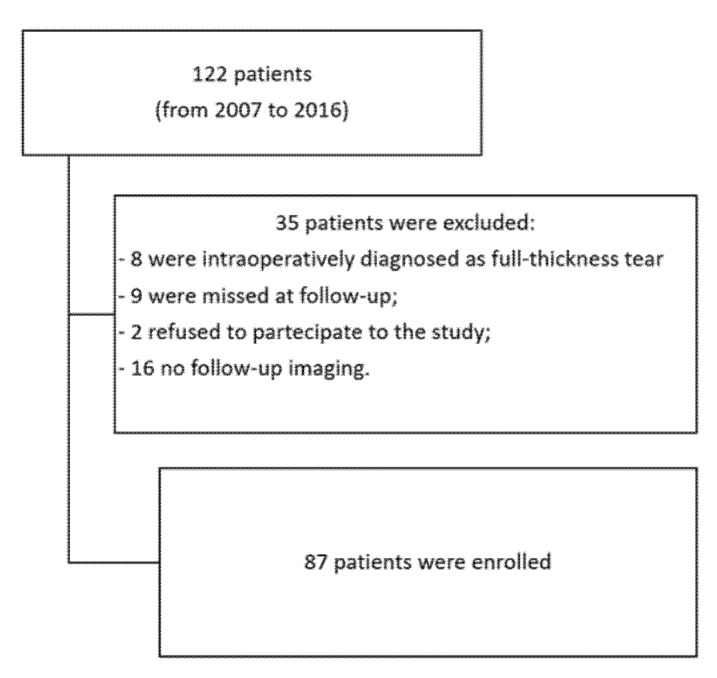
Flowchart of the inclusion process.

Eight patients were excluded as they had a full-thickness tear diagnosed intraoperatively despite preoperative MRI evidence of a partial lesion; nine patients were lost during the follow-up; two patients declined to participate in the study for personal reasons; postoperative MRI or ultrasound assessment was not available for sixteen patients (Figure 5).

Therefore, eighty-seven patients (87 shoulders, 43 men, 44 women) were included in the study. Patient median age was 57 years (range 18–73), and the median follow-up was 5 years (range 2–7). The majority of the lesions occurred on the dominant side (60.9%), with the main cause of PT-RCTs being tendon degeneration (70.1%), as it was macroscopically confirmed during arthroscopic procedures. According to the Ellman classification, PT-RCTs were classified as articular (61, 70.1%), bursal (17, 19.6%) and intratendinous (9, 10.3%). The median time to surgery was 5 months (range 2–11), while the median time to return to work was 90 days (range 30–365) (Table 1).

Regarding LHBT disorders, a tenodesis was performed in active young patients (21, 24.1%). In patients from 50 to 65 years of age, a tenodesis to the coracohumeral ligament was carried out (54, 62.1%). In older patients, a tenotomy of LHBT was performed (12, 13.8%).

**Figure 6:**
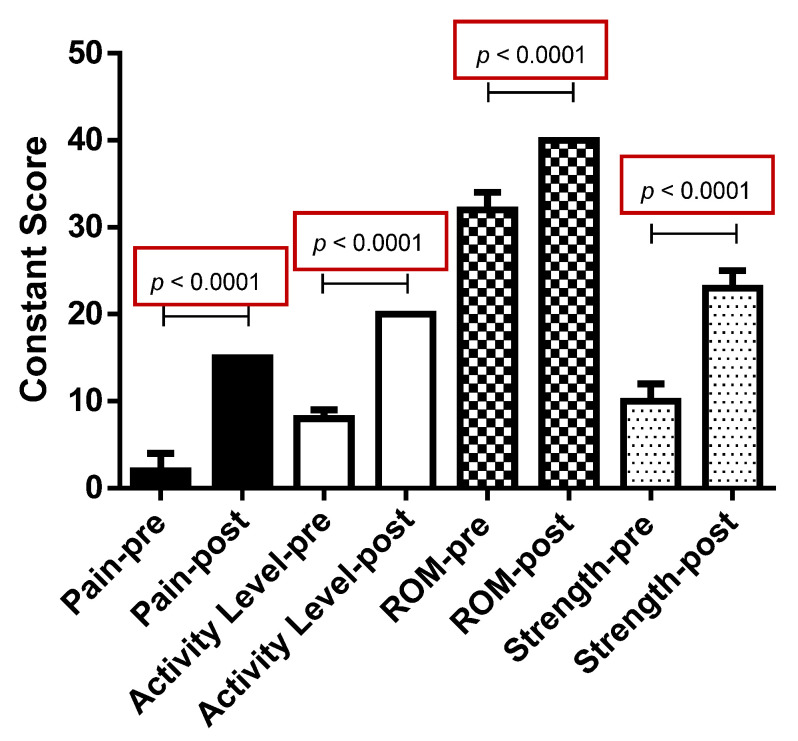
Bar graph showing the improvement of all the domains of the Constant Score after the TCR technique: pain, activity level, range of motion (ROM), and strength. TCR, tear completion repair; ROM, range of movement.

Results showed significant improvement of the total CS and each domains of pain, daily living activities, active ROM, and strength after surgery (Figure 6; Table 2).

The median preoperative CS was 53.5, while postoperative CS was 94.0 (*p* < 0.0001) with a gain of 40 points. Median improvement of pain, daily living activities, active ROM and strength was 10.5, 11, 6 and 12 points, respectively. The VAS pain score was significantly improved from 8.6 to 1.0 (*p* < 0.0001). The median value for patient satisfaction was 10 (range 4–10); 85 patients (97.7%) were highly satisfied with the overall improvement in pain and functional recovery, while two patients (2.3%) who had developed postoperative adhesive capsulitis, were not satisfied with the outcome. A weak correlation was observed between pre- and postoperative CS (*r* = 0.252, *p* = 0.019). No correlations were found among BMI, age, patient satisfaction and CS. No correlations were found between time to surgery, follow-up, and CS.

Patients were divided into three groups according to the Elmann classification, and their demographics and clinical outcomes were compared (Table 3). No differences were found regarding age, BMI, and clinical outcomes among the groups (Table 3).

Patients were also divided by sex. The two groups (males vs. females) were comparable in terms of age and BMI (*p* = 0.540 and *p* = 0.110, respectively). The preoperative CS of women (median 51.5 [23–67.5]) was lower compared to that of men (median 55 [34–68.5]) (*p* = 0.038), while the postoperative CS between the two groups was borderline (*p* = 0.060). The preoperative VAS was higher in women (median 10 [3–10]) than in men (median 8 [4–10]) (*p* = 0.016), while the postoperative VAS was lower in women (median 1 [0–5]) compared to men (median 1.7 [0–5]) (*p* = 0.039). No differences were found in ROM except for preoperative passive IR 1, postoperative active, and passive IR 1 (*p* = 0.02, *p* = 0.014, *p* = 0.005, respectively). No differences were found regarding patient satisfaction between women and men (*p* = 0.914).

Patients were divided into two groups depending on age, group 1 patients with age < 60 (*n* = 47) and group 2 patients with age ≥ 60 (*n* = 40), to investigate the influence of aging on clinical outcomes. No differences were reported (*p* > 0.05).

Active smokers (14.9%) were compared with non-smokers observing no difference in both preoperative and postoperative clinical scores. All patients were advised to stop smoking 4 weeks before and at least 6 weeks after surgery, to monitor dyslipidemia and glycemia, and to take medical therapy.

Seven (8.0%) cases of postoperative adhesive capsulitis were observed. No other major complications were observed.

One patient required revision surgery as he presented with a complete retear of supraspinatus tendon due to an accidental trauma (bike fall). Four patients (4.6%) experienced a partial retear diagnosed with MRI or ultrasound, which did not affect functional outcome.

## 4. Discussion

The present study shows that arthroscopic TCR of PT-RCT, with “critical zone” removal and microfractures, results in good clinical outcomes with low complication rates after a median of 5 years post-surgery independent from sex, age, and smoking.

Specifically, patients showed improvement in the CS domains of pain, daily living activities, active ROM and strength after surgery, with low failure rates and only a small number of postoperative adhesive capsulitis. Pain and strength showed the best improvements.

The retear rate after arthroscopic repair of PT-RCT ranges from 5% to 12% [36,37,38]. In our cohort, 5 patients presented with a retear (5.7%), of which only one required revision surgery due to a complete traumatic retear after a bike fall. Four patients experienced asymptomatic partial retears diagnosed at follow-up by MRI or ultrasound, which did not affect functional outcome and did not require any treatment.

In addition, 7 (8.0%) cases developed postoperative adhesive capsulitis that were successfully managed with steroid injection and physical therapy without affecting the outcome, in agreement with the literature [39].

In our study, smoking and comorbidities did not affect the clinical outcomes, but there were few patients who smoked and few with comorbidities. No differences were found dividing patients depending by age, and no correlations with postoperative constant score were observed. The preoperative Constant score of women was found to be lower compared to that of men but this difference was not observed at the follow-up. Interestingly, women had a higher preoperative VAS but a lower postoperative VAS.

Some authors previously reported that tear location influenced the outcomes. Weber et al. and Cordasco et al. described worse results in high grade bursal-side tears treated with debridement and subacromial decompression (SAD) [40,41]. However, in high-grade bursal-side lesions, tendon quality is highly compromised as a result of the inflammatory insult caused by the subacromial bursitis and the friction between the tendon surface and the acromial arch [42]. Therefore, debridement and SAD alone may result insufficient for such lesions because they do not allow the removal of the pathological portion of the tendon. Hence, the impact of the residual tendinitis of the supraspinatus could explain the worse results reported by these studies compared to our data [40,41].

Some authors adopt different surgical approaches depending on the thickness of the lesion, preferring debridement with or without SAD for Ellman grade I and II tears and tendon repair in grade III lesions [23,43]. The effectiveness of debridement plus SAD is still debated. Budoff et al. published good to excellent results after arthroscopic rotator cuff debridement without decompression in 60 patients with Ellman grade I, II and even III lesions, with only 4% of the cohort needing revision surgery for a secondary full-thickness tear [43]. Conversely, Kartus et al. reported negative long-term outcomes after arthroscopic SAD in patients with PT-RCT, showing a progression to a complete tear at 5 years in the 34.6% of cases [44].

In our cohort, isolated debridement and SAD without tendon repair were not performed—not even for grade I and II tears—because pain and functional impairment are not solely determined by subacromial bursitis and impingement but also by tendon rupture.

The superiority of TTR or TCR for PT-RCT is still controversial as both techniques have yielded good results [27,28,45]. Kim et al. compared TTR and TCR techniques in a prospective randomised controlled study reporting no differences in functional outcome but a significantly higher retear rate for bursal-side tears in TCR repair (23.3% vs. 3.3%) [27]. In contrast, Jordan et al. showed lower incidence of postoperative stiffness and pain, and higher functional recovery of TCR compared to the TTR technique [39].

Park et al. suggested the adoption of different arthroscopic treatments depending on tear size, recommending TTR for tears greater than 50% of tendon thickness and TCR when they extend beyond 90% [26].

Even if tear location and size may have a significant role in tendon healing, the severity of the supraspinatus tendinosis represents the main cause of failure [42]. Tendinosis is thought to be caused by repetitive microtears in the tendon tissue, involving both collagen breakdown and vascular alteration [46]; microtears are more frequent in PT-RCT compared to FT-RCT [42]. This is because PT-RCTs are characterised by a tension mismatch between the inner and outer layers of the RC, which is not present in complete tears [42]. In addition, there is often a severe subacromial bursitis in PT-RCTs, particularly extensive in superficial tears, which can worsen the inflammation and consequently tendon healing [47].

It should be considered that the granulation tissue, formed at the healing site and composed of new connective tissue and blood vessels, is poorly differentiated and has weak mechanical properties, which could facilitate retearing [48,49,50]. In addition, intrinsic degenerative changes in the repaired tendon will continue if the diseased tendon is reinserted. Therefore, a complete removal of the pathological and scarcely vascularised tendon tissue, such as performed by the TCR technique, is preferable to facilitate tendon healing process. The etiology of RC tendinopathy is related to a deficient vascular supply of the RC tendons [17,50], even though it is still debated in the literature [51].

Codman first described the “critical zone”, an area of decreased vascularity within the supraspinatus tendon about 1 cm medial to its insertion on the greater tuberosity, which is most commonly involved in RCT [30]. Recently, several cadaveric studies have examined the blood supply of RC, identifying a hypovascular zone at the articular surface of the tendon, just medial to the footprint [52,53,54]. Moreover, an in vivo study demonstrated a statistically significant reduction of microcirculation at the edge of a degenerative RC lesion [55], according to Codman. The TCR technique allows a complete debridement of the “critical zone”, improving chances of tendon healing and decreasing the rate of tear progression [17,54,55,56,57,58].

After tendon edge removal with our technique, the footprint is cleared from the residual tendon tissue and gently decorticated. Then microfractures, the so-called Crimson duvet, are performed as biological support, improving the chances of tendon healing [31].

Finally, acromioplasty is performed because it offers multiple advantages, such as improved visualisation of the subacromial space, resolution of impingement symptoms and release of growth and angiogenic factors from the acromion [59,60,61].

The aims of RC repair are not only to achieve better initial fixation strength and good mechanical stability but also to obtain biological healing of the tendon-to-bone interface, hampering the formation of scar tissue [62]. Several biological strategies have been suggested to promote tendon healing including growth factors, cell therapy, scaffolds, and tissue engineering [62,63]. Even though some techniques have shown promising results, their clinical use is still limited. Different bone marrow stimulation techniques, such as “microfractures”, “multiple channeling”, “microvascularisation”, and “bone venting” of the greater tuberosity, have been used to promote RC tendon healing with promising clinical outcomes [63]. Inflammatory cells present in the haematoma—neutrophils, macrophages and platelets—begin releasing cytokines and growth factors in the injury area. These substances initiate the next phase, stimulating cell proliferation and migration of tenocytes, angiogenesis and chemoattraction of additional inflammatory cells. These inflammatory cells release mediators which upregulate proliferation of tenocytes from undamaged tendon fragments and recruit fibroblasts from adjacent structures [64].

Milano et al. compared single-row repair with and without microfracture in a clinical prospective randomised study, reporting a higher healing rate in the microfracture group for chronic large-to-massive tears [65]. Bielsel et al. reported the results of an experimental study with New Zealand rabbits, showing the effectiveness of microfractures in the healing process of RCTs [63].

The present study has some limitations: first, the retrospective nature of the study and second, the absence of a comparison with other surgical techniques. Moreover, most of the patients had articular ruptures, making the comparison with bursal and intratendinous unbalanced. However, it is well-known that bursal and intratentinous ruptures are uncommon. Finally, although all arthroscopic procedures were performed over a long period (2007–2016), we believe our results could not have been affected by the learning curve of the experienced surgeon (the senior author), specialised in shoulder surgery, who has been performing about 350/year arthroscopic procedures from 1997 until September 2020, the year of his retirement. Hence, his surgical experience probably reached peak efficiency before starting patient recruitment.

The strengths of this study are the enrollment of a large cohort of patients with well-defined PT-RTCs treated by the same senior surgeon with a highly standardised technique. Importantly, tendon healing was confirmed by MRI or ultrasound. Furthermore, this study includes intratendinous tears, which are poorly described in the literature.

## 5. Conclusions

Patients with PT-RCTs of the supraspinatus tendon treated by the TCR technique showed good functional results, excellent recovery of strength, and a high degree of patient satisfaction with good resolution of painful symptoms even at mid-term follow-up, regardless of tear location.

An accurate debridement of the “critical zone,” associated with biological stimulation of tendon healing by microfractures and a stable fixation, could be crucial in promoting tendon healing by improving the biological and biomechanical properties of the repaired tissue.

## Figures and Tables

**Table 1:** Main characteristics of the cohort.

Variable	Value (*n* = 87)
Age (years)	56.0 ± 9.5; 57.0 [18.0–73.0]
Sex, number (%)	
Female Male	44 (50.6%) 43 (49.4%)
BMI	25.0 ± 2.9; 24.9 [18.7–33.3]
Time (months) to surgery	5.1 ± 1.6; 5.0 [2.0–11.0]
Follow-up (years)	4.7 ± 1.4; 5.0 [2.0–7.1]
Side of involvement	
D, number (%): ND, number (%)	53 (60.9%): 34 (39.1)
Traumatic onset, number (%)	26 (29.9%)
Corticosteroid injections	20 (23.0%)
Tear location (Ellman classification) number (%)	
articular bursal intratendinous	61 (70.1%) 17 (19.6%) 9 (10.3%)
Comorbidities, number (%)	
Diabetes Hypertension Dyslipidemia Osteoporosis	5 (5.7%) 19 (21.8%) 23 (26.3%) 5 (5.7%)
Smoking, number (%)	13 (14.9%)
Capsulitis, number (%)	7 (8%)
Return to work, days	110.85 ± 70.25; 90 [30–365]
Satisfaction	9.26 ± 1.28; 10 [4–10]

SD = standard deviation, D = dominant, ND = non-dominant. All variables are reported as mean ± SD; median [min-max], except for categorical ones expressed as number and percentage.

**Table 2:** Pre- and postoperative visual analog scale (VAS) pain and Constant Score.

	Preoperative	Postoperative (Last Follow-Up)	*p* Value
VAS pain (mean ± SD; median [min-max])	8.6 ± 1.6; 9.0 [3.0–10.0]	0.9 ± 1.3; 0 [0–5.0]	<0.0001
Constant score (mean ± SD; median [min-max])	53.5 ± 9.0; 53.5 [23.0–68.5]	91.3 ± 11.0; 94.0 [37.5–100.0]	<0.0001
Pain (mean ± SD; median [min-max])	2.5 ± 1.9; 2.0 [0–7.5]	12.7 ± 2.9; 15.0 [4.0–15.0]	<0.0001
Activity level (mean ± SD; median [min-max])	8.6 ± 2.9; 8.0 [0–17.0]	18.7 ± 2.9; 20.0 [8.0–20.0]	<0.0001
ROM (mean ± SD; median [min-max])	32.6 ± 4.5; 32.0 [16.0–40.0]	38.0 ± 3.9; 40.0 [14.0–40.0]	<0.0001
Strength (mean ± SD; median [min-max])	9.7 ± 3.1; 10.0 [4.0–18.0]	21.7 ± 4.2; 23.0 [8.0–25.0]	<0.0001

VAS = Visual Analogue Scale, SD = standard deviation, ROM = range of movement.

**Table 3:** Demographics and clinical outcomes dividing patients according to the Elmann classification of anatomical location of the lesion.

	Articular (*n* = 61)	Bursal (*n* = 17)	Intratendinous (*n* = 9)	*p*-Value
Age (years) (mean ± SD; median [min-max])	56.2 ± 10.3; 59 [18.0–73.0]	56.6 ± 7.6; 56.0 [45.0–72.0]	53.4 ± 7.9; 53.0 [40.0–67.0]	n.s
Sex, number (%)				0.038
Females Males	34 (55.7) 27 (44.3)	4 (56.4) 13 (76.5)	6 (66.7) 3 (33.3)	
Traumatic onset, number (%)				n.s
Yes No	12 (70.6) 5 (29.4)	41 (67.2) 20 (32.8)	8 (88.9) 1 (11.1)	
BMI (mean ± SD; median) [min-max])	25.1 ± 3.1; 24.9 [18.7–33.3]	24.8 ± 2.2; 24.6 [21.5–28.4]	24.9 ± 2.8; 24.7 [20.8–29.5]	n.s
Return to work				n.s
(mean ± SD; median) [min-max]	115.3 ± 74.3; 90.0 [30.0–365.0]	98.8 ± 71.2; 80.0 [40.0–365.0]	103.3 ± 30.8; 90.0 [60.0–150.0]	
Satisfaction				n.s
(mean ± SD; median) [min-max]	9.1 ± 1.4; 10.0 [4.0–10.0]	9.5 ± 0.9; 10.0 [7.0–10.0]	9.7 ± 0.5; 10.0 [9.0–10.0]	
Constant score pre-				n.s
(mean ± SD; median) [min-max]	53.4 ± 8.9; 53.0 [28.0–68.0]	56.2 ± 5.8; 58.0 [40.5–65.0]	49.0 ± 13.4; 52.5 [23.0–68.5]	
Constant score post-				n.s
(mean ± SD; median) [min-max]	90.7 ± 12.4; 94.0 [37.5–100.0]	95.0 ± 4.5; 95.0 [83.0–100.0]	88.6 ± 7.7; 92.0 [75.0–100.0]	
VAS pain pre-				n.s
(mean ± SD; median) [min-max]	8.7 ± 1.5; 9.0 [4.0–10.0]	7.9 ± 1.5; 8.0 [5.0–10.0]	8.9 ± 2.3; 10.0 [3.0–10.0]	
VAS pain post-				n.s
(mean ± SD; median) [min-max]	1.0 ± 1.2; 0 [0–5.0]	0.8 ± 1.5; 0 [0–5.0]	1.3 ± 1.4; 1.0 [0–3.0]	

SD = standard deviation, BMI = Body Mass Index, VAS = Visual Analogue Scale. n.s. = non-significant.

## Data Availability

The dataset generated during the current study is available from the corresponding authors on reasonable request.

## References

[1] Ellman H. (1990). Diagnosis and treatment of incomplete rotator cuff tears. Clin. Orthop. Relat. Res..

[2] Strauss E.J., Salata M.J., Kercher J., Barker J.U., McGill K., Bach B.R., Romeo A.A., Verma N.N. (2011). The arthroscopic management of partial-thickness rotator cuff tears: A systematic review of the literature. Arthroscopy.

[3] Sher J.S., Uribe J.W., Posada A., Murphy B.J., Zlatkin M.B. (1995). Abnormal findings on magnetic resonance images of asymptomatic shoulders. J. Bone Jt. Surg. Am..

[4] Matava M.J., Purcell D.B., Rudzki J.R. (2005). Partial-thickness rotator cuff tears. Am. J. Sports Med..

[5] Cigolotti A., Biz C., Lerjefors E., de Iudicibus G., Belluzzi E., Ruggieri P. (2020). Medium- to long-term clinical and functional outcomes of isolated and combined subscapularis tears repaired arthroscopically. Arch. Med. Sci..

[6] Connor P.M., Banks D.M., Tyson A.B., Coumas J.S., D’Alessandro D.F. (2003). Magnetic resonance imaging of the asymptomatic shoulder of overhead athletes: A 5-year follow-up study. Am. J. Sports Med..

[7] Fukuda H. (2000). Partial-thickness rotator cuff tears: A modern view on Codman’s classic. J. Shoulder Elb. Surg..

[8] Sano H., Ishii H., Trudel G., Uhthoff H.K. (1999). Histologic evidence of degeneration at the insertion of 3 rotator cuff tendons: A comparative study with human cadaveric shoulders. J. Shoulder Elb. Surg..

[9] Milgrom C., Schaffler M., Gilbert S., van Holsbeeck M. (1995). Rotator-cuff changes in asymptomatic adults. The effect of age, hand dominance and gender. J. Bone Jt. Surg. Br..

[10] Factor D., Dale B. (2014). Current concepts of rotator cuff tendinopathy. Int. J. Sports Phys..

[11] Chambler A.F., Pitsillides A.A., Emery R.J. (2003). Acromial spur formation in patients with rotator cuff tears. J. Shoulder Elb. Surg..

[12] Levigne C., Garret J., Grosclaude S., Borel F., Walch G. (2012). Surgical technique arthroscopic posterior glenoidplasty for posterosuperior glenoid impingement in throwing athletes. Clin. Orthop. Relat. Res..

[13] Zabrzyński J., Huri G., Gryckiewicz S., Çetik R.M., Szwedowski D., Łapaj Ł., Gagat M., Paczesny Ł. (2020). Biceps Tenodesis versus Tenotomy with Fast Rehabilitation Protocol-A Functional Perspective in Chronic Tendinopathy. J. Clin. Med..

[14] Varacallo M., Seaman T.J., Mair S.D. (2020). Biceps Tendon Dislocation and Instability. StatPearls.

[15] Lazarides A.L., Alentorn-Geli E., Choi J.H., Stuart J.J., Lo I.K., Garrigues G.E., Taylor D.C. (2015). Rotator cuff tears in young patients: A different disease than rotator cuff tears in elderly patients. J. Shoulder Elb. Surg..

[16] Fukuda H. (2003). The management of partial-thickness tears of the rotator cuff. J. Bone Jt. Surg. Br..

[17] Seitz A.L., McClure P.W., Finucane S., Boardman N.D., Michener L.A. (2011). Mechanisms of rotator cuff tendinopathy: Intrinsic, extrinsic, or both?. Clin. Biomech..

[18] Ogawa K., Yoshida A., Inokuchi W., Naniwa T. (2005). Acromial spur: Relationship to aging and morphologic changes in the rotator cuff. J. Shoulder Elb. Surg..

[19] Clavert P., Le Coniat Y., Kempf J.F., Walch G. (2016). Intratendinous rupture of the supraspinatus: Anatomical and functional results of 24 operative cases. Eur. J. Orthop. Surg. Traumatol..

[20] Lee S.B., Nakajima T., Luo Z.P., Zobitz M.E., Chang Y.W., An K.N. (2000). The bursal and articular sides of the supraspinatus tendon have a different compressive stiffness. Clin. Biomech..

[21] Yamanaka K., Matsumoto T. (1994). The joint side tear of the rotator cuff. A followup study by arthrography. Clin. Orthop. Relat. Res..

[22] Lo I.K., Denkers M.R., More K.D., Nelson A.A., Thornton G.M., Boorman R.S. (2018). Partial-thickness rotator cuff tears: Clinical and imaging outcomes and prognostic factors of successful nonoperative treatment. Open Access J. Sports Med..

[23] Oliva F., Piccirilli E., Bossa M., Via A.G., Colombo A., Chillemi C., Gasparre G., Pellicciari L., Franceschetti E., Rugiero C. (2015). IS Mu. LT—Rotator Cuff Tears Guidelines. Muscles Ligaments Tendons J..

[24] Biz C., Vinanti G.B., Rossato A., Arnaldi E., Aldegheri R. (2012). Prospective study of three surgical procedures for long head biceps tendinopathy associated with rotator cuff tears. Muscles Ligaments Tendons J..

[25] Liem D., Alci S., Dedy N., Steinbeck J., Marquardt B., Mollenhoff G. (2008). Clinical and structural results of partial supraspinatus tears treated by subacromial decompression without repair. Knee Surg. Sports Traumatol. Arthrosc..

[26] Park J.Y., Yoo M.J., Kim M.H. (2003). Comparison of surgical outcome between bursal and articular partial thickness rotator cuff tears. Orthopedics.

[27] Kim Y.S., Lee H.J., Bae S.H., Jin H., Song H.S. (2015). Outcome Comparison Between in Situ Repair Versus Tear Completion Repair for Partial Thickness Rotator Cuff Tears. Arthroscopy.

[28] Rossi L.A., Atala N.A., Bertona A., Bongiovanni S., Tanoira I., Maignon G., Ranalletta M. (2019). Long-Term Outcomes After In Situ Arthroscopic Repair of Partial Rotator Cuff Tears. Arthroscopy.

[29] Vap A.R., Mannava S., Katthagen J.C., Horan M.P., Fritz E.M., Pogorzelski J., Millett P.J. (2018). Five-Year Outcomes After Arthroscopic Repair of Partial-Thickness Supraspinatus Tears. Arthroscopy.

[30] Codman E.A. (1934). The Shoulder: Rupture of the Supraspinatus Tendon and Other Lesions in or About the Subacromial Bursa.

[31] Snyder S.J., Burns J. (2009). Rotator cuff healing and the bone marrow “crimson duvet” from clinical observations to science. Tech. Shoulder Elb. Surg..

[32] Baumgarten K.M., Chang P.S., Foley E.K. (2019). Patient-determined outcomes after arthroscopic rotator cuff repair with and without biceps tenodesis utilizing the PITT technique. J. Shoulder Elb. Surg..

[33] Sekiya J.K., Elkousy H.A., Rodosky M.W. (2003). Arthroscopic biceps tenodesis using the percutaneous intra-articular transtendon technique. Arthroscopy.

[34] Neer C.S. (1972). Anterior acromioplasty for the chronic impingement syndrome in the shoulder: A preliminary report. J. Bone Jt. Surg. Am..

[35] Aguado G., Obando D.V., Herrera G.A., Ramirez A., Llinas P.J. (2019). Retears of the Rotator Cuff: An Ultrasonographic Assessment During the First Postoperative Year. Orthop. J. Sports Med..

[36] Kamath G., Galatz L.M., Keener J.D., Teefey S., Middleton W., Yamaguchi K. (2009). Tendon integrity and functional outcome after arthroscopic repair of high-grade partial-thickness supraspinatus tears. J. Bone Jt. Surg. Am..

[37] Franceschi F., Papalia R., Del Buono A., Vasta S., Costa V., Maffulli N., Denaro V. (2013). Articular-sided rotator cuff tears: Which is the best repair? A three-year prospective randomised controlled trial. Int. Orthop..

[38] Kim K.C., Shin H.D., Cha S.M., Park J.Y. (2014). Repair integrity and functional outcome after arthroscopic conversion to a full-thickness rotator cuff tear: Articular- versus bursal-side partial tears. Am. J. Sports Med..

[39] Jordan R.W., Bentick K., Saithna A. (2018). Transtendinous repair of partial articular sided supraspinatus tears is associated with higher rates of stiffness and significantly inferior early functional scores than tear completion and repair: A systematic review. Orthop. Traumatol. Surg. Res..

[40] Cordasco F.A., Backer M., Craig E.V., Klein D., Warren R.F. (2002). The partial-thickness rotator cuff tear: Is acromioplasty without repair sufficient?. Am. J. Sports Med..

[41] Weber S.C. (1999). Arthroscopic debridement and acromioplasty versus mini-open repair in the treatment of significant partial-thickness rotator cuff tears. Arthroscopy.

[42] Chung S.W., Kim J.Y., Yoon J.P., Lyu S.H., Rhee S.M., Oh S.B. (2015). Arthroscopic repair of partial-thickness and small full-thickness rotator cuff tears: Tendon quality as a prognostic factor for repair integrity. Am. J. Sports Med..

[43] Budoff J.E., Rodin D., Ochiai D., Nirschl R.P. (2005). Arthroscopic rotator cuff debridement without decompression for the treatment of tendinosis. Arthroscopy.

[44] Kartus J., Kartus C., Rostgard-Christensen L., Sernert N., Read J., Perko M. (2006). Long-term clinical and ultrasound evaluation after arthroscopic acromioplasty in patients with partial rotator cuff tears. Arthroscopy.

[45] Nathani A., Smith K., Wang T. (2018). Partial and Full-Thickness RCT: Modern Repair Techniques. Curr. Rev. Musculoskelet. Med..

[46] Sharma P., Maffulli N. (2006). Biology of tendon injury: Healing, modeling and remodeling. J. Musculoskelet. Neuronal Interact..

[47] Bonutti P.M., Hawkins R.J. (1989). Rotator cuff disorders. Bailliere Clin. Rheumatol..

[48] Jensen P.T., Lambertsen K.L., Frich L.H. (2018). Assembly, maturation, and degradation of the supraspinatus enthesis. J. Shoulder Elb. Surg..

[49] Ju Y.J., Muneta T., Yoshimura H., Koga H., Sekiya I. (2008). Synovial mesenchymal stem cells accelerate early remodeling of tendon-bone healing. Cell Tissue Res..

[50] Karthikeyan S., Griffin D.R., Parsons N., Lawrence T.M., Modi C.S., Drew S.J., Smith C.D. (2015). Microvascular blood flow in normal and pathologic rotator cuffs. J. Shoulder Elb. Surg..

[51] Hegedus E.J., Cook C., Brennan M., Wyland D., Garrison J.C., Driesner D. (2010). Vascularity and tendon pathology in the rotator cuff: A review of literature and implications for rehabilitation and surgery. Br. J. Sports Med..

[52] Brooks C.H., Revell W.J., Heatley F.W. (1992). A quantitative histological study of the vascularity of the rotator cuff tendon. J. Bone Jt. Surg. Br..

[53] Lin T.W., Cardenas L., Soslowsky L.J. (2004). Biomechanics of tendon injury and repair. J. Biomech..

[54] Rudzki J.R., Adler R.S., Warren R.F., Kadrmas W.R., Verma N., Pearle A.D., Lyman S., Fealy S. (2008). Contrast-enhanced ultrasound characterization of the vascularity of the rotator cuff tendon: Age- and activity-related changes in the intact asymptomatic rotator cuff. J. Shoulder Elb. Surg..

[55] Biberthaler P., Wiedemann E., Nerlich A., Kettler M., Mussack T., Deckelmann S., Mutschler W. (2003). Microcirculation associated with degenerative rotator cuff lesions. In vivo assessment with orthogonal polarization spectral imaging during arthroscopy of the shoulder. J. Bone Jt. Surg. Am..

[56] Fukuda H., Hamada K., Yamanaka K. (1990). Pathology and pathogenesis of bursal-side rotator cuff tears viewed from en bloc histologic sections. Clin. Orthop. Relat. Res..

[57] Goodmurphy C.W., Osborn J., Akesson E.J., Johnson S., Stanescu V., Regan W.D. (2003). An immunocytochemical analysis of torn rotator cuff tendon taken at the time of repair. J. Shoulder Elb. Surg..

[58] Matthews T.J., Hand G.C., Rees J.L., Athanasou N.A., Carr A.J. (2006). Pathology of the torn rotator cuff tendon. Reduction in potential for repair as tear size increases. J. Bone Jt. Surg. Br..

[59] Kim K.C., Lee W.-Y., Shin H.D., Joo Y.-B., Han S.-C., Chung H.-J. (2019). Repair integrity and functional outcomes of arthroscopic repair for intratendinous partial-thickness rotator cuff tears. J. Orthop. Surg..

[60] Randelli P., Margheritini F., Cabitza P., Dogliotti G., Corsi M.M. (2009). Release of growth factors after arthroscopic acromioplasty. Knee Surg. Sports Traumatol. Arthrosc..

[61] Galliera E., Randelli P., Dogliotti G., Dozio E., Colombini A., Lombardi G., Cabitza P., Corsi M.M. (2010). Matrix metalloproteases MMP-2 and MMP-9: Are they early biomarkers of bone remodelling and healing after arthroscopic acromioplasty?. Injury.

[62] Lorbach O., Baums M.H., Kostuj T., Pauly S., Scheibel M., Carr A., Zargar N., Saccomanno M.F., Milano G. (2015). Advances in biology and mechanics of rotator cuff repair. Knee Surg. Sports Traumatol. Arthrosc..

[63] Bilsel K., Yildiz F., Kapicioglu M., Uzer G., Elmadag M., Pulatkan A., Esrefoglu M., Bozdag E., Milano G. (2017). Efficacy of bone marrow-stimulating technique in rotator cuff repair. J. Shoulder Elb. Surg..

[64] Zabrzyński J., Łapaj Ł., Paczesny Ł., Zabrzyńska A., Grzanka D. (2018). Tendon—function-related structure, simple healing process and mysterious ageing. Folia Morphol..

[65] Milano G., Saccomanno M.F., Careri S., Taccardo G., De Vitis R., Fabbriciani C. (2013). Efficacy of marrow-stimulating technique in arthroscopic rotator cuff repair: A prospective randomized study. Arthroscopy.

